# Global trends in migraine and anxiety over the past 10 years: a bibliometric analysis

**DOI:** 10.3389/fneur.2024.1448990

**Published:** 2025-01-22

**Authors:** Biao Huang, Weining Chen, Chunmei Peng, Yu Wang, Xiuli Shen, Qi Zhang, Liu Yang, Jun Wu

**Affiliations:** ^1^Department of Acupuncture, Affiliated Hospital of Jiangxi University of Traditional Chinese Medicine, Nanchang, China; ^2^Graduate School, Jiangxi University of Traditional Chinese Medicine, Nanchang, China; ^3^Department of Asset Management, Jiangxi University of Traditional Chinese Medicine, Nanchang, China; ^4^Department of Gastroenterology, Affiliated Hospital of Jiangxi University of Traditional Chinese Medicine, Nanchang, China; ^5^Medical Department, Affiliated Hospital of Jiangxi University of Traditional Chinese Medicine, Nanchang, China

**Keywords:** migraine, anxiety, global research trends, bibliometric analysis, visualization

## Abstract

**Background:**

Recent studies have shown that migraine significantly increases the incidence of anxiety and is positively correlated with the severity and frequency of migraine. The relationship between migraine and anxiety has attracted extensive attention. This study focused on the association between migraine and anxiety, aiming to predict potential future research trends.

**Methods:**

A bibliometric analysis was conducted using publications from the Core Collection of Web of Science. We utilized CiteSpace.5.8.R3 and VOSviewer 1.6.17 to evaluate the value of articles over the past 10 years.

**Results:**

The number of publications has increased significantly over the past 10 years. The cooperative network analysis shows that the United States is the most collaborative country. Additionally, Harvard University is the institution and Richard B. Lipton the individual with the highest number of studies on migraine. The analysis of keyword outbreaks indicates that the strong citation burst words are closely related to sex differences, activation, allodynia, and preventive treatment, which represent emerging new research areas and potential hotspots for future research.

**Conclusion:**

An overall upward trend in the research of migraine and anxiety was observed. Sex differences, functional magnetic resonance imaging (fMRI), activation, allodynia, and preventive treatment are predicted to be hotspots in the future.

## 1 Introduction

Migraine is a common neurological disorder that has remained one of the major health issues affecting the quality of life and daily functioning of patients (1). Severe headaches that afflict both men and women and recur at different frequencies are the representative features of migraine (2). According to comprehensive assessment of research studies, the anticipated worldwide lifetime prevalence of migraine was 17.5% for both genders, 21.0% for women, and 11.6% for men (3). According to the Global Burden of Disease 2019 report, migraine ranks top among the health issues affecting women under 50 years of age and the second overall in terms of causes of disability (4).

Clinical studies have found that long-term migraine patients are prone to mental and emotional problems such as anxiety, depression, and insomnia, and these patients have poor prognosis and are prone to recurrence (5, 6). The incidence of anxiety and depression in migraine patients is 2–10 times higher than that of the general population, greatly affecting their quality of life (7). Migraine and emotional disorders, such as anxiety and depression, coexist (8). Compared to individuals with depression or anxiety alone or without both, individuals with serious depressive and anxiety disorders together are more likely to experience migraines (9).

## 2 Materials and methods

### 2.1 Data collection and retrieval strategies

**Figure 1:**
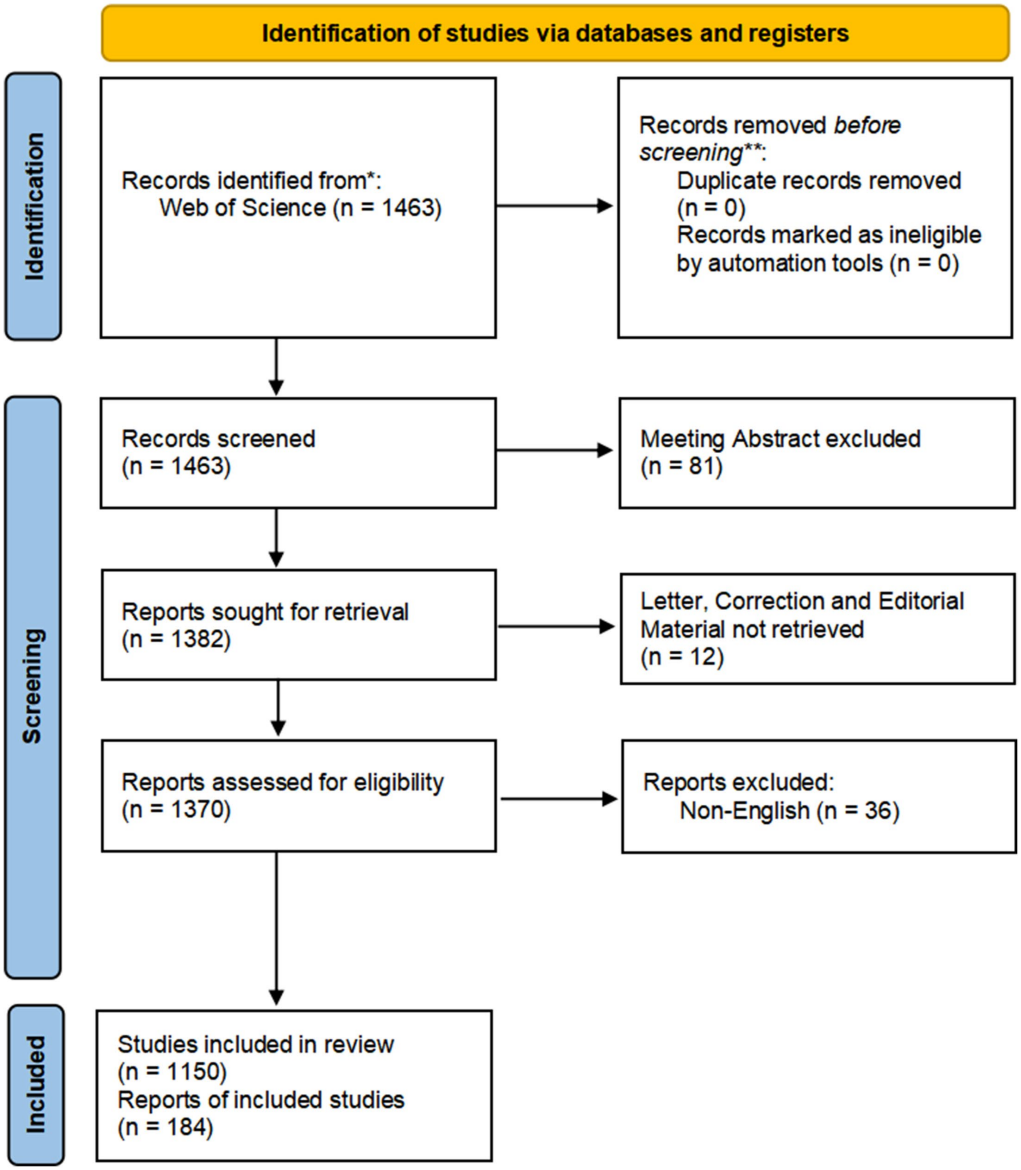
Flowchart of the study.

We selected the Web of Science Core Collection (WoSCC) from Clarivate Analytics as the retrieval database for this study. The WoSCC is a highly authoritative citation index database that is widely used in scientific research and bibliometric studies. We used a combination of subject words and free words for line search. The subject words included migraine and anxiety. The free words mainly included migraine and anxiety. We set the search language as “English.” The search document type was set to “Article or Review.” We set the publication date condition of the retrieved literature as “2012 to 2021.” The retrieval process was carried out independently by two researchers on 5 May 2022. The detailed results of this retrieval are shown in Figure 1.

### 2.2 Data analysis

The software packages used for data analysis in this study are Microsoft Office Excel 2021, CiteSpace.5.8.R3, and Vosviewer1.6.17. Microsoft Office Excel 2021 was used for the statistics of literature years and the production of related tables. CiteSpace.5.8.R3 was used to analyze the number of published countries, institutions, and authors in the data, to examine their collaboration relationships, and to generate the keyword occurrence map and keyword burst map. Vosviewer1.6.17 was used to analyze highly cited and co-cited references in the data. This study utilizes WoSCC to analyze the publication output, subject category, publication year, author, and other functions. Then, the selected documents were imported into CiteSpace, and the time span was 2012–2021. The node was selected based on the type of analysis to be performed. The specific parameter settings in CiteSpace were as follows: Time slicing: January 2012–December 2021; Term source: Title, Abstract, Author Keywords, and Keyword Plus; Node type: Author, Institution, Country, and Keyword; Link strength: Cosine; and Selection Criteria: Top N = 50. In the generated map, the color of the circular nodes represents the time when the article was published. The thickness of the node is positively related to the frequency. The thickness of the line describes the strength between the projects, and the color of the line describes the year when the two projects first collaborated. The nodes in the map represent elements such as author, country/region, or institution. The link lines between the nodes indicate the collaboration relationship. The larger the circle, the greater the number of articles that were published. The wider the line, the stronger the relationship. The outermost purple ring represents the centrality. The centrality value represents the cooperation intensity, and the higher the value, the stronger the cooperation. VOSviewer software was used to statistically analyze highly cited and co-cited documents. First, the results obtained were imported through WOS into software. Then, the number of terms to be selected was set to 10. Finally, the results were imported to Excel for sorting.

Bibliometrics is a research investigation assessment methodology that has been used extensively. By using both quantitative and qualitative scientific analytical approaches, researchers can investigate detailed information on trends in the growth of related subjects (10). It is utilized to analyze many aspects of publications in a certain topic, including data and contributions about countries, organizations, authors, journals, keywords, and references (11). Numerous studies have demonstrated the potential association and pathophysiological mechanisms between migraine and anxiety (12–15). However, no specific investigation has yet been conducted on the knowledge mapping of migraine and anxiety. Thus, this research was aimed at comprehensively analyzing the knowledge base and emerging trends on migraine and anxiety.

## 3 Results

### 3.1 Literature screening results

According to the literature search strategy, a total of 1,463 articles were retrieved. Titles, abstracts, and even the full texts of articles were used to filter out articles that are closely related to our subject. Only 1,334 publications were selected for the review. Detailed retrieval results are shown in Figure 1.

### 3.2 Analysis of publication trends

**Figure 2:**
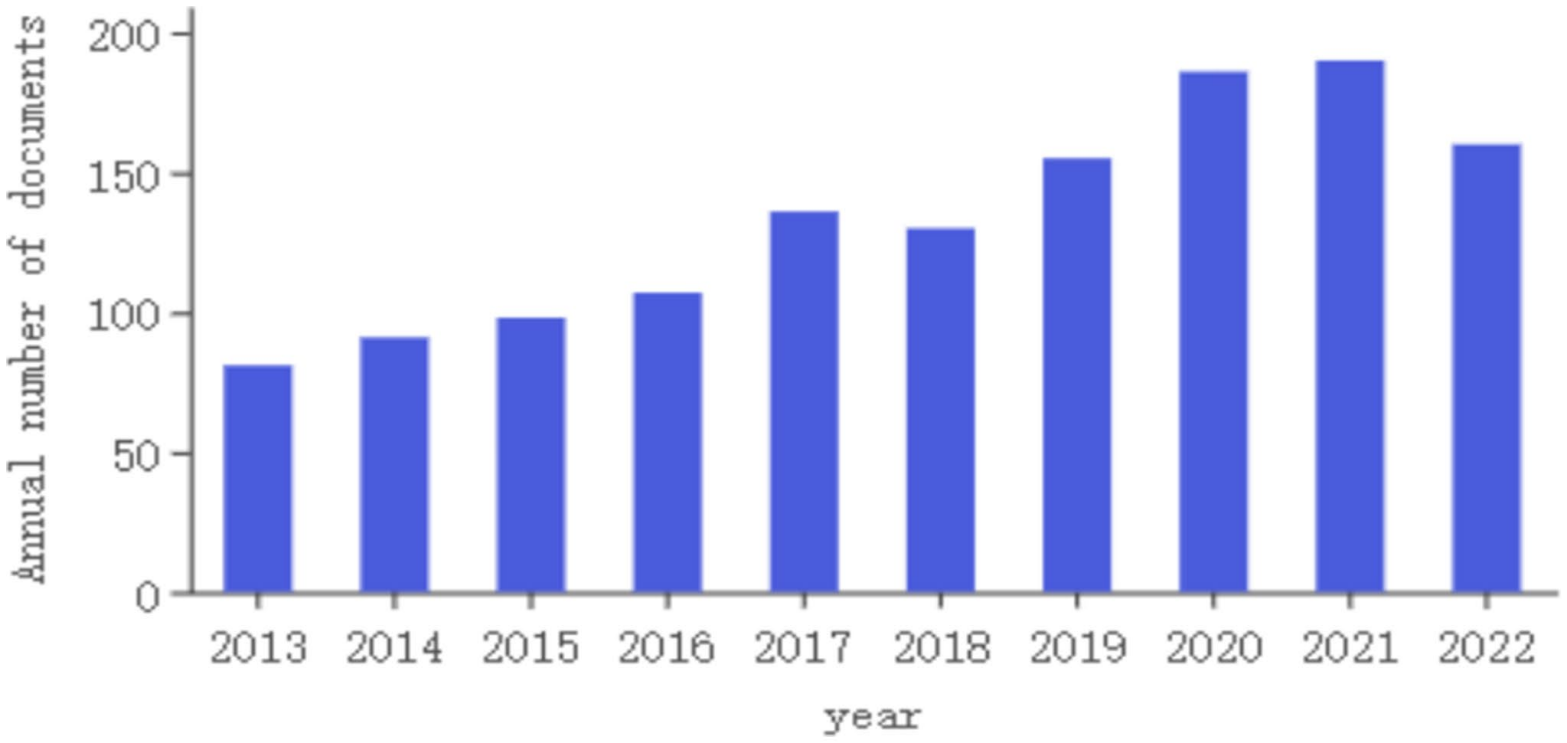
Number of publications per year.

As shown in Figure 2, the number of articles published in 2013 was at least 81, and the number of articles published in 2021 was at most 190. From 2013 to 2022, the annual publications on migraine and anxiety have consistently increased, indicating that this field has become more popular and attracted increasing attention.

### 3.3 Analysis of the contribution of major countries

A total of 109 countries participated in research on migraine and anxiety from 2013 to 2022. Table 1 lists the top 10 countries that have conducted research on migraine and anxiety over the past 10 years. The United States had the highest number of publications (431), followed by Italy (158), and China (103). Italy, Britain, Germany, France, Spain, and Turkey are all European countries, while China is the only Asian country represented in this research. Both the United States and Canada are located in North America, while Brazil is located in South America. In terms of centrality, England ranked first among 10 countries (0.13), showing its global influence in the study of migraine and anxiety.

**Table 1:** The 10 countries with the most publications.

Ranking	Countries	Centrality	Year	Publications
1	United States	0.10	2013	431
2	Italy	0.04	2013	158
3	China	0.01	2013	103
4	England	0.13	2013	97
5	Germany	0.03	2013	89
6	Spain	0.03	2013	85
7	Turkey	0.00	2013	85
8	Canada	0.03	2013	68
9	Brazil	0.00	2013	62
10	France	0.03	2013	59

### 3.4 Analysis of major institutions

**Figure 3:**
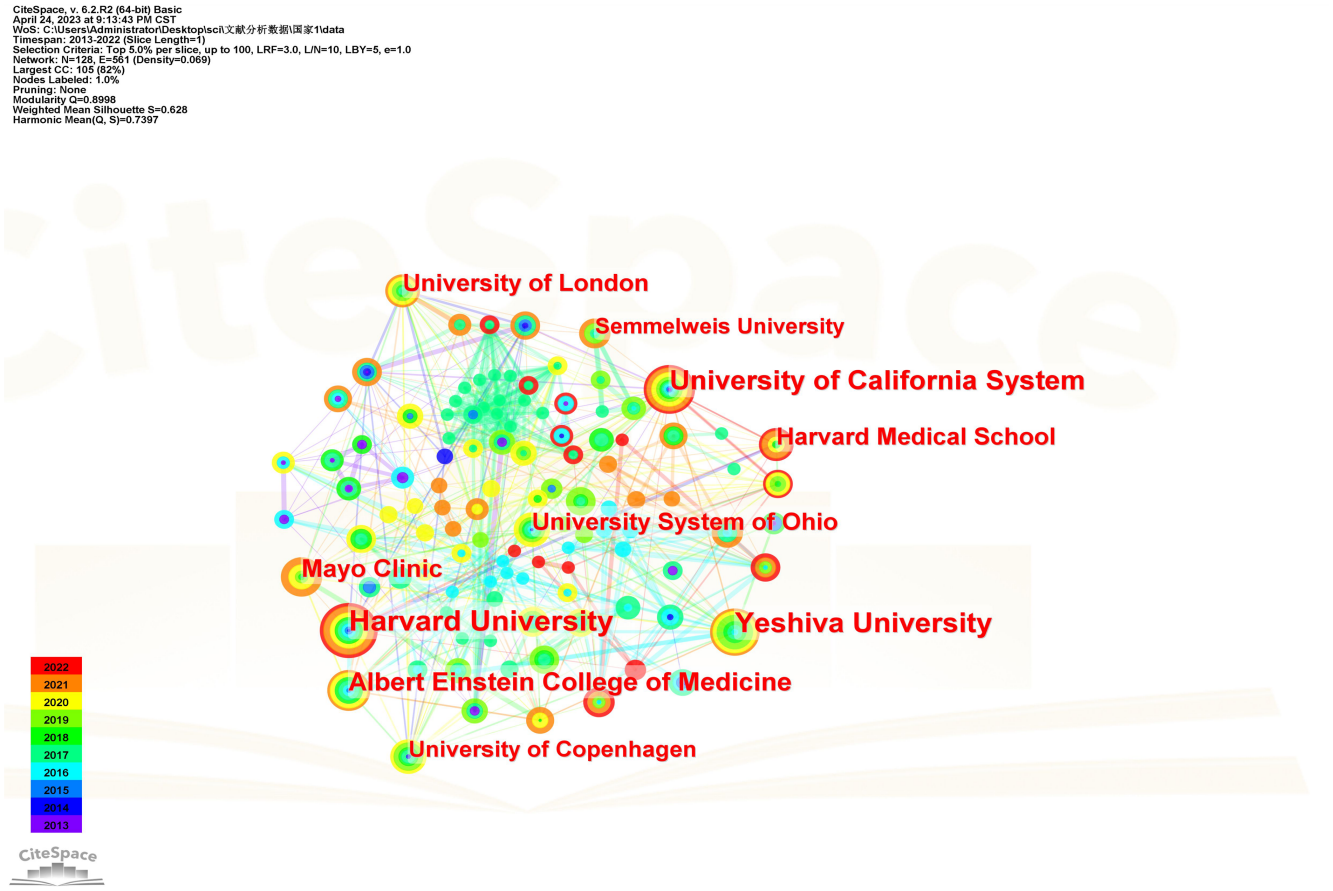
Institutional cooperation network.

From 2013 to 2022, a total of 320 institutions participated in migraine and anxiety research. Table 2 shows the top 10 institutions. Of these, institutions with a publication volume greater than 35 articles include Harvard University (*n* = 59), Yeshiva University (*n* = 50), University of California System (*n* = 46), Albert Einstein College of Medicine (*n* = 38), and Mayo Clinic (*n* = 36). Figure 3 shows the collaborative network between major publishing institutions in this topic. The greater the centrality, the stronger the representativeness.

**Table 2:** Top 10 institutions with the most publications.

Rank	Institution	Centrality	Year	Publications
1	Harvard University	0.16	2013	59
2	Yeshiva University	0.07	2013	50
3	University of California System	0.11	2013	46
4	Albert Einstein College of Medicine	0.02	2013	38
5	Mayo Clinic	0.07	2013	36
6	University System of Ohio	0.04	2013	34
7	Harvard Medical School	0.09	2014	33
8	University of London	0.13	2014	29
9	University of Copenhagen	0.07	2014	24
10	Semmelweis University	0.02	2016	24

### 3.5 Analysis of the main authors

**Figure 4:**
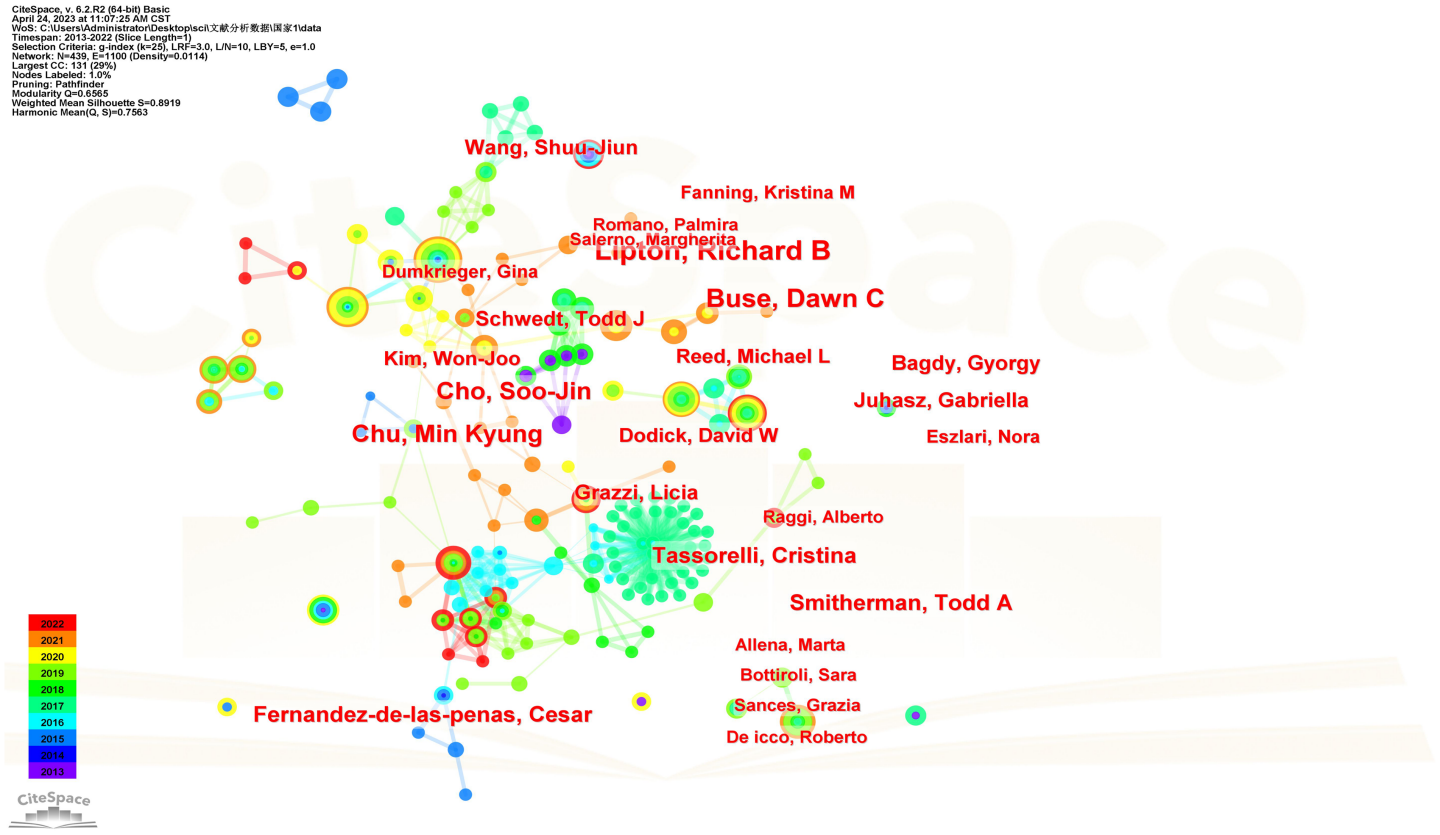
The network of relationships between the main authors.

Over the past 10 years, a total of 433 authors participated in the study of migraine and anxiety. Table 3 shows the top 10 authors. Richard B. Lipton (*n* = 30), Buse Dawn C (*n* = 24), Chu Min Kyung (*n* = 21), and Cho Soo-Jin (*n* = 21) each published over 20 articles. Figure 4 depicts the network of relationships between the main authors in this field. Among them, Richard B. Lipton and Chu Min Kyung have collaborated. Bochnia Kuster, Joao Guilherme, and Tan. Bin Cheng worked together. Additionally, Cho, Soo Jin, and Chu Min Kyung also collaborated with one another.

**Table 3:** The 10 authors with the highest number of articles.

Ranking	Author	Centrality	Year	Publications
1	Lipton, Richard B	0.06	2013	30
2	Buse, Dawn C	0.01	2013	24
3	Chu, Min Kyung	0	2013	21
4	Cho, Soo-Jin	0	2013	21
5	Tassorelli,Cristina	0.1	2013	15
6	Fernandez-de-las-penas, Cesar	0	2013	15
7	Smitherman,ToddA	0	2013	14
8	Schwedt, Todd j	0.01	2013	12
9	Bagdy, Gyorgy	0	2013	12
10	Wang, Shuu-Jiun	0	2013	12

### 3.6 Keyword analysis

**Figure 5:**
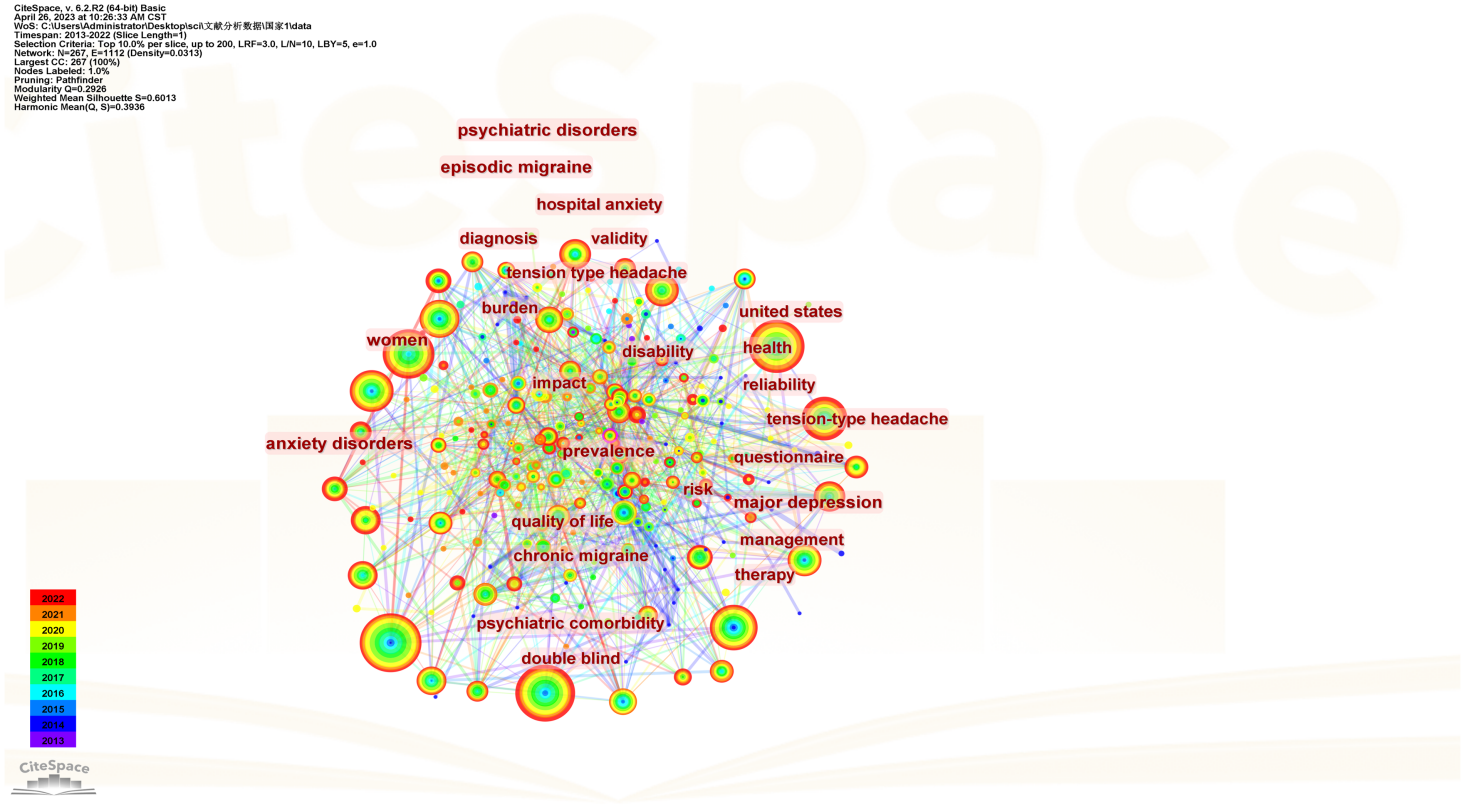
Keyword co-occurrence visualization.

**Figure 6:**
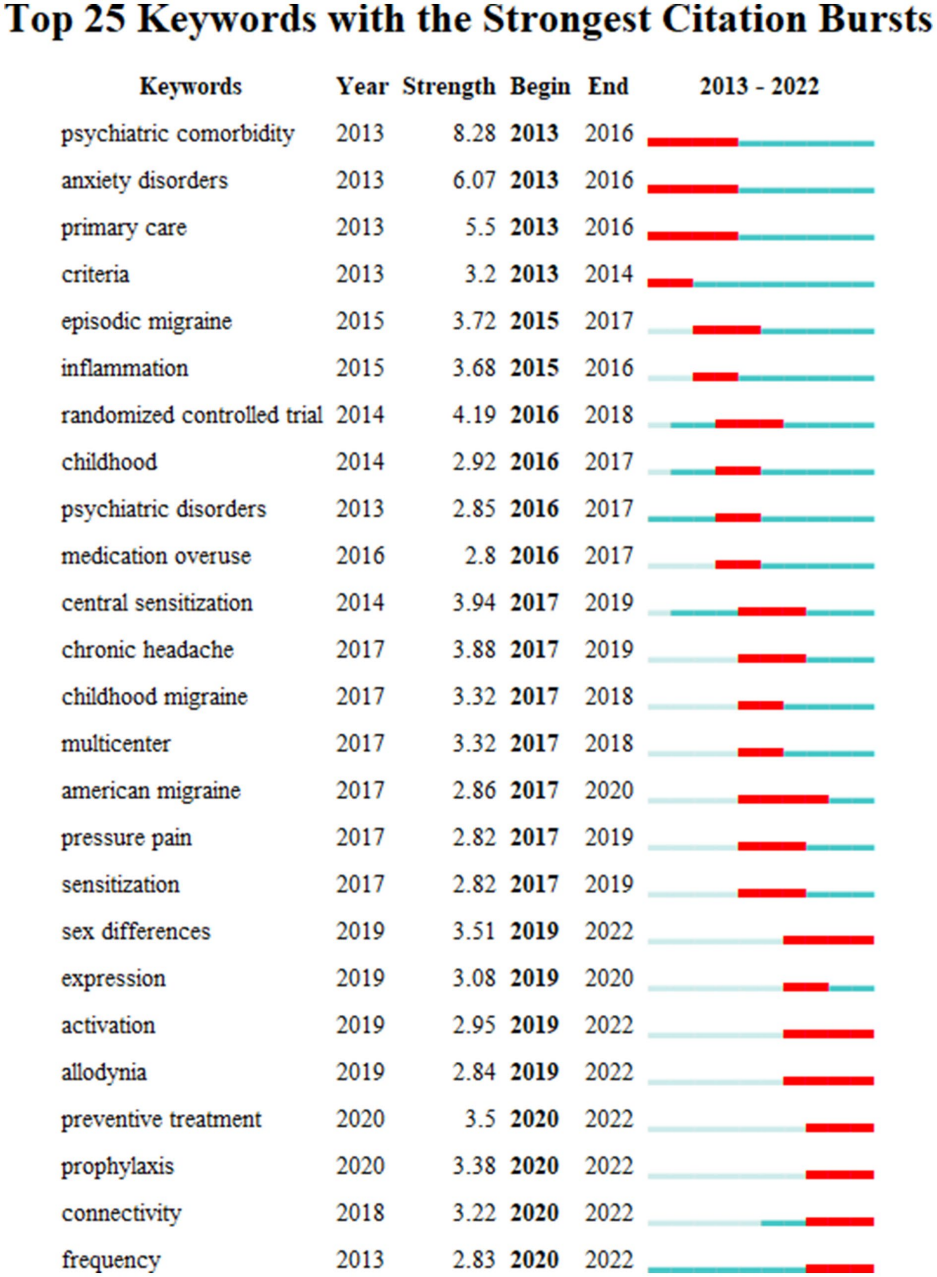
Keyword burst chart.

**Figure 7:**
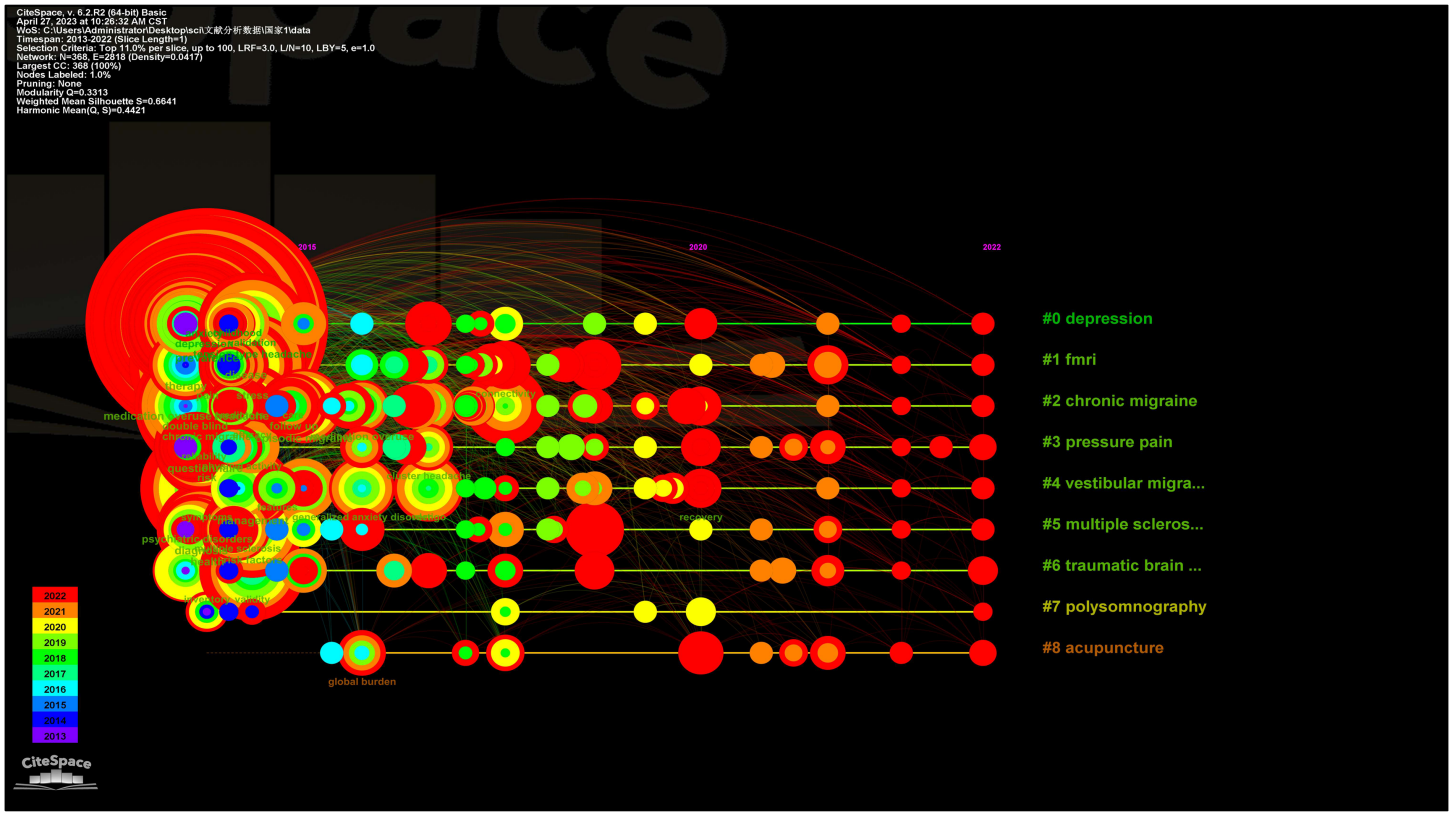
Keyword clustering timeline graph.

From 2013 to 2022, 460 keywords were found in 1,334 articles on migraine and anxiety. Table 4 lists the top 20 terms sorted by frequency in this field in Table 4. The terms “anxiety,” “depression,” “migraine,” and “headache” are four diseases with a frequency greater than 200. Figure 5 displays the co-occurrence network of keywords with a frequency surpassing 25 in studies related to migraine and anxiety. The node size represents the frequency of the keywords, and the connection between nodes represents two keywords appearing simultaneously in the same article. “Prevalence,” “anxiety,” “depression,” “migraine,” “headache,” and “quality of life” were high-frequency keywords, indicating that these terms were closely related research topics. As shown in Figure 6, 25 items with the highest prevalence are listed. Since 2019, sex difference, expression, activation, and allodynia have emerged as significant keywords in this field. Figure 7 illustrates the correlation between keyword clustering and time. The nine major keyword groups related to migraine and anxiety research are “#0 depression,” “#1 fmri,” “#2 chronic migraine,” “#3 pressure pain,” “#4 vestibular migraine,” “#5 multiple sclerosis,” “#6 traumatic brain injury,” “#7 polysomnography,” and “#8 acupuncture.”

**Table 4:** The 20 keywords with the highest frequency.

Ranking	Keywords	Centrality	Year	Count
1	Prevalence	0.05	2013	331
2	Anxiety	0.02	2013	290
3	Depression	0.01	2013	290
4	Migraine	0.02	2013	280
5	Headache	0.02	2013	207
6	Quality of life	0.05	2013	193
7	Disorders	0.05	2013	165
8	Disability	0.03	2013	148
9	Pain	0.02	2014	145
10	Chronic migraine	0.02	2013	122
11	Symptoms	0.01	2013	120
12	Impact	0.02	2014	109
13	Symptoms	0.02	2013	107
14	Population	0	2013	100
15	Association	0.04	2015	95
16	Epidemiology	0.05	2013	92
17	Children	0.03	2013	91
18	Psychiatric comorbidity	0.04	2013	90
19	Chronic pain	0.03	2013	86
20	Double blind	0.04	2013	74

### 3.7 Analysis of high-yielding journals

Table 5 lists the top 10 journals published on this topic. *Headache* (*n* = 123), *Journal of Headache and Pain* (*n* = 103), *Cephaalgia* (*n* = 64), *Frontiers in Neurology* (*n* = 47), and *Neurological Sciences* (*n* = 34) are journals that have published more than 30 articles. In addition, *Headache* (*n* = 2,647), *Journal of Headache and Pain* (*n* = 2,787), and *Cephaalgia* (*n* = 1,161) all have citations greater than 1,000 times.

**Table 5:** Top 10 journals with the most publications.

Ranking	Journal	Citations	Publications
1	HEADACHE	2,647	132
2	JOURNAL OF HEADACHE AND PAIN	2,787	103
3	CEPHALALGIA	1,161	64
4	FRONTIERS IN NEUROLOGY	494	47
5	NEUROLOGICAL SCIENCES	378	34
6	CURRENT PAIN AND HEADACHE REPORTS	280	20
7	JOURNAL OF AFFECTIVE DISORDERS	166	16
8	PAIN MEDICINE	342	16
9	INTERNATIONAL JOURNAL OF ENVIRONMENTAL RESEARCH AND PUBLIC HEALTH	48	15
10	JOURNAL OF NEUROLOGY	446	15

### 3.8 Analysis of highly cited articles

During the period of 2013 to 2022, a total of 1,334 articles were published on migraine and anxiety. Among them, three studies have been cited over 500 times. Table 6 shows the top 10 articles with the highest number of citations in this field. Three of them have been cited in over 1,000 publications.

**Table 6:** Ten highly cited articles.

Rank	Title	Journals	First author	Year	Citations
1	Global, regional, and national incidence, prevalence, and years lived with disability for 310 diseases and injuries, 1990–2015: a systematic analysis for the Global Burden of Disease Study 2015	LANCET	Vos Theo	2016	2,754
2	Diagnostic Criteria for Temporomandibular Disorders (DC/TMD) for Clinical and Research Applications: Recommendations of the International RDC/TMD Consortium Network and Orofacial Pain Special Interest Group	JOURNAL OF ORAL & FACIAL PAIN AND HEADACHE	Schiffman Eric	2014	1,784
3	Global, regional, and national incidence, prevalence, and years lived with disability for 328 diseases and injuries for 195 countries, 1990–2016: a systematic analysis for the Global Burden of Disease Study 2016	LANCET	Vos Theo	2017	1,484
4	Global, regional, and national burden of neurological disorders during 1990–2015: a systematic analysis for the Global Burden of Disease Study 2015	LANCET NEUROLOGY	Feigin Valery L	2017	410
5	Comorbidities of epilepsy: current concepts and future perspectives	LANCET NEUROLOGY	Keezer Mark R	2016	320
6	Sex differences in the epidemiology, clinical features, and CrossMark pathophysiology of migraine	LANCET NEUROLOGY	Vetvik kjersti Grotta	2017	273
7	Effect of Fremanezumab Compared With Placebo for Prevention of Episodic Migraine A Randomized Clinical Trial	JAMA-JOURNAL OF THE AMERICAN MEDICAL ASSOCIATION	Dodick David W	2018	259
8	Management of psychiatric and neurological comorbidities in epilepsy	NATURE REVIEWS NEUROLOGY	Kanner Andres MM	2016	211
9	Migraine and its psychiatric comorbidities	JOURNAL OF NEUROLOGY NEUROSURGERY AND PSYCHIATRY	Minen, Mia Tova	2016	205
10	Psychiatric comorbidities of episodic and chronic migraine	JOURNAL OF NEUROLOGY	Buse, Dawn C	2013	205

## 4 Discussion

### 4.1 General information

In this study, we used CiteSpace 58.R3 and VOSviewer 1.6.17 to evaluate the value of articles over the past 10 years. Since 2013, a significant number of academic achievements showed a growing frequency of migraine and anxiety research, with 109 countries, 320 institutions, 433 authors, and 1,334 journals publishing articles. Over the past 10 years, the United States had published the highest number of research articles on migraine and anxiety among all countries, while the country with the highest centralization was the United Kingdom. We found that Richard B. Lipton was the notable contributor with a high scholarly influence on the field. However, it is obvious that many researchers lack cooperation and communication with other scholars. Harvard University, Yeshiva University, University of California System, Albert Einstein College of Medicine, and Mayo Clinic were considered central institutions in the field with high publications. Additionally, we also noticed that institutions are closely interconnected and engage in extensive collaboration. “Prevalence,” “anxiety,” “depression,” “migraine,” “headache,” and “quality of life” were closely related to the topic of research. The keyword timeline graph suggests that research on functional magnetic resonance imaging (fMRI) is an significant topic in this field. Finally, research on acupuncture has emerged as a significant area in this field after 2015 and continues to be prominent.

### 4.2 Frontier and hotspots

Keywords with red lines extending into the latest year may indicate future research frontiers. Figure 6 shows the top 25 keywords with the strongest citation bursts. Sex differences, activation, allodynia, preventive treatment, prophylaxis, connectivity, and frequency were the keywords presenting us with the main areas of current migraine and anxiety research.

#### 4.2.1 Sex differences

It was reported that there existed a significant interaction between anxiety and depression in relation to the development of migraines, influenced by age and gender (16). From the study of women’s health across the nation, it was found that migraine was associated with an increased prevalence of anxiety in midlife women (17). One cross-sectional study concluded that anxiety significantly predicts the occurrence of migraines (18), and the female gender was a significant predictor of anxiety, with women being 7.2 times more likely to experience anxiety compared to men. The majority of people with migraines were women, which may be related to estrogen levels (19). The occurrence of migraine can be influenced by menarche, menstruation, pregnancy, menopause, usage of hormonal contraceptives, and hormone replacement therapy. These changes are caused by fluctuations in estrogen levels, which have an impact on the cerebral vasculature or cellular excitability (19). In addition, the drop of estrogen levels may be a trigger for migraine in women and the activation of the NLRP3 inflammasome in the hippocampus caused by estrogen deficiency has been demonstrated in animal studies to contribute to anxiety-like behavior in female rodents (16). The above results showed that hormone levels might be a potential mechanism of the sex differences in migraine and anxiety. However, these findings require confirmation in prospective studies.

#### 4.2.2 Preventive treatment

It is possible for anxiety and/or depression to precipitate migraine episodes, and those who have frequent occurrences and those with chronic migraine are more likely to experience psychological distress (20). Previously, it has been proposed that migraine and psychological comorbidities may have a common pathophysiology with unique pathophysiological pathways that overlap or interact (20). In patients with migraine, preventive (prophylactic) treatment includes the use of prescription drugs such as beta-blockers, antidepressants, antiepileptics, calcium-channel blockers, and anti-CGRP mAbs (21, 22). The purpose of preventive treatment is to reduce the frequency, length, or intensity of episodes. The choice of medicine, response to preventative medication, behavioral migraine therapy, and compliance with migraine treatment programs can all be affected by co-occurring depression and/or anxiety (23). Amitriptyline and nortriptyline are tricyclic antidepressants (TCAs) that have been proven to be effective in treating episodic migraines (24). TCAs have been shown to prevent migraines by inhibiting serotonin and norepinephrine reuptake as well as antagonizing the 5-HT2 receptor. To prevent episodic migraines, greater dosages of TCAs may be necessary to treat the underlying depression in patients with comorbid depression (25). Monoclonal antibody treatments targeting calcitonin gene-related peptide (CGRP) are used widely as preventive migraine pharmacotherapies. It has been identified as a neuropeptide that plays a critical role in the pathophysiology of migraines, both centrally and peripherally (26). The innovative preventative medicines for migraine that target the CGRP system have an impact on anxiety, depressed symptoms, and exhaustion in addition to improving the intensity of the disease (9). Patients with comorbid depression and migraine may benefit from fremanezumab as it can lower disability and lessen the frequency of migraine and headache days (27). Compared to fremanezumab, galcanezumab and erenumab appear to be more successful in treating concurrent depression and anxiety symptoms in migraine patients. To completely comprehend the effect of CGRP antagonists on mental health conditions linked to migraines, it is helpful to analyze the results of validated questionnaires such as PHQ-9, HDRS, BDI-II, HARS, and GAD-7 (28). However, because of their novelty, the effectiveness of CGRP antagonists in treating comorbid psychiatric patients is limited (29). In addition, in order to avoid diminished quality of life and lower the burden of migraines, preventative medication should be initiated early in the course of the disease. In Europe, there is a critical need for access to migraine preventive treatment that is both effective and comfortable (30).

#### 4.2.3 Allodynia

The location of central sensitization (CS) in migraine is generally considered to be the spinal trigeminal nucleus, which receives convergent input from the dura and the face (31). Migraine-associated allodynia, which is defined as a nociceptive reaction to ordinary harmless stimuli, involves the activation of the trigeminal system and development of central sensitization (32). A cross-sectional study showed that reduced pressure pain threshold, an inhibitory prognostic profile, and impaired somatosensory function were observed in the migraine group. These patients had a high level of central sensitization, mild anxiety symptoms, and pain catastrophizing as part of their psychosocial profile (33). It was proposed that headache chronification is caused by shared neural pathways between anxiety and depression (34). A major risk factor leading to the trigeminal nerve system’s hyperexcitability is long-term, unregulated stress and anxiety (35). One study found that chronic exposure to secondary environmental stress caused persistent alterations in the expression of proteins related to the development of peripheral and central sensitization in the trigeminal nerve (36), including elevated levels of CGRP and GFAP as well as the active forms of the MAP kinases ERK, p38, and JNK. These modifications all contribute to and sustain the trigeminal nerve system’s hyperexcited condition (36). Moreover, in patients with migraine, excessive occupation of the NMDA receptor due to elevated glutamatergic system activity may intensify and reinforce pain transmission, as well as cause allodynia and central sensitization (37).

#### 4.2.4 fMRI

The fMRI technique has the advantages of high spatial resolution, no radiation, and non-invasiveness, which enable researchers to measure structural and functional brain changes non-invasively in patients with migraine or anxiety/depression (38, 39). One of the hot topics in study was the functional connectivity of brain regions. The top 10 brain regions where depressed patients exhibited significant differences from healthy controls were the Amygdala_L, Insula_R, Frontal_Inf_Oper_R, Cingulum_Post_L, Putamen_L, Thalamus_R, Angular_L, Precuneus_R, Frontal_Sup_R, and Occipital_Inf_L, according to a bibliometrics study and meta-analysis (40). Brain regions shown to have altered functional connectivity (FC) in patients with migraine include those that participate in sensory-discriminative processing of pain (e.g., the somatosensory cortex and posterior insula regions), affective processing (e.g., the anterior insula and amygdala), cognitive processing (e.g., the prefrontal cortex and hippocampus), and pain modulation (e.g., the periaqueductal gray) (41). Clinical studies have shown that the degree of anxiety for patients with chronic pain is closely related to the activation of specific brain regions and connectivity of resting brain networks (42). During migraine attacks, DMN has increased functional connectivity in pain-related brain regions such as the thalamus, insula, and left posterior central gyrus (43). A previous fMRI study investigated the abnormal activity of the left medial prefrontal cortex (mPFC) in people with migraine and depression comorbidities (44). The intrinsic activity of the left mPFC was activated in patients with depression, and the activity of the left mPFC was also increased in patients with migraine (44). As the anterior node of the DMN, the mPFC had high activation at rest but had increased activity during cognitive and emotional processing. Moreover, ESN, PPN, and VN were involved in the neuropathological mechanisms of co-occurrence of migraine and depression (45).

### 4.3 Limitations

First, in the process of literature retrieval, the newly published literature and some keywords may not have been retrieved and included in the statistical analysis, and the results may be affected by incomplete literature collection. Second, this study only retrieved data from WoSCC, ignoring other databases, so some articles may have been missed. Finally, solely English publications were incorporated, which potentially introduced the impact of non-English scholarly contributions.

## 5 Conclusion

In this study, we utilized CiteSpace.5.8.R3 and VOSviewer 1.6.17 to evaluate the value of articles over the past 10 years. Since 2013, there has been an overall upward trend in the number of publications on migraine and anxiety research, with 109 countries, 320 institutions, 433 authors, and 1,334 journals publishing articles. However, international cooperation is expected in the future. Sex differences, activation, allodynia, and preventive treatment will be emerging research areas and potential hotspots for future research. In conclusion, this article provides the recent research status and research hotspots and frontiers in this field, delivering a perspective on the progressive direction of migraine and anxiety research, which may be valuable for further study of this field in the future.

## Data Availability

The original contributions presented in the study are included in the article/supplementary material, further inquiries can be directed to the corresponding author.
